# The fertile grounds of reproductive activism in The Gambia: A qualitative study of local key stakeholders’ understandings and heterogeneous actions related to infertility

**DOI:** 10.1371/journal.pone.0226079

**Published:** 2019-12-04

**Authors:** Susan Dierickx, Gily Coene, Megan Evans, Julie Balen, Chia Longman

**Affiliations:** 1 Centre of Expertise on Gender, Diversity and Intersectionality (RHEA), Vrije Universiteit Brussel, Brussel, Belgium; 2 Centre for Research on Culture and Gender, Ghent University, Ghent, Belgium; 3 School of Health and Related Research, The University of Sheffield, Sheffield, United Kingdom; University of Cape Coast, GHANA

## Abstract

**Introduction:**

While several studies have focussed on the experiences of women living with infertility, there is a paucity of information related to understandings, representations and actions of key stakeholders (i.e. organisations and individual actors involved in activities or professional care surrounding infertility) when it comes to infertility in Sub-Saharan Africa. This ethnographic study conducted in The Gambia, West Africa, focuses on how key stakeholders in the country understand infertility, and on their activities to improve the lives of people with infertility.

**Methodology:**

This ethnographic study draws on primary and secondary data for thematic analysis. Primary qualitative data were collected using in-depth interviews, observations, informal conversations and group discussion with various stakeholders (i.e. health care providers and representatives of non-governmental, governmental and international organisations). Sources of secondary data included government and non-governmental reports and media outputs.

**Results:**

Results illustrated that most key stakeholders had a good understanding of the cultural frameworks and social realities of women living with infertility, with less focus on, or awareness of, men’s experiences of infertility. We distinguished three different positions of these actors and organisations, first, the infertility supporters, i.e. those who despite political challenges and a lack of funding, initiated activities to raise awareness about the problems people with infertility are facing and aim to increase access to infertility services. The second are moderate supporters, i.e. those who recognise the problems infertility poses and whose organisations target some of the perceived causes of infertility (i.e. lack of health education and harmful cultural practices). A third group of neutral or moderate opponents consist mainly of formal health care providers who do not consider infertility a current priority, given many competing demands in the resource-constrained healthcare system.

**Conclusion:**

While international donors still largely neglect the emotional and social implications of infertility in Sub-Saharan African countries, some local stakeholders are working to bring services closer to people with infertility. The efforts of these local stakeholders require support and integration, and should include engaging with different groups for widespread sensitisation to reduce stigma and promote attendance to health centres for reproductive health challenges.

## Introduction

The 1994 International Conference on Population and Development (ICPD) held in Cairo was a key moment for the progress towards more equitable sexual and reproductive health and rights [1–4]. The new global aim identified during the ICPD was to enable all people to have ‘the capability to reproduce and the freedom to decide if, when and how often to do so’ (UNFPA, paragraph 7.2). This holistic approach towards reproductive health was translated into the ICPD’s Program of Action, recommending that reproductive health services should incorporate treatment and prevention of infertility. Following the ICPD, infertility was recognised as a priority within several declarations and policy documents of international organisations and meetings (e.g. the World Conference on Women (1995), the World Health Assembly (2004), the World Summit (2005) and the World Health Organisation Global Health Strategy (2011)). However, many international donor organisations fail to consider infertility as a priority. For example, the most recent ‘ICPD Beyond 2014’ Program of Action does not mention infertility care for women, let alone men who are largely missing from the ICPD document except as potential detriments to the health of women [5,6]. Several scholars and activists have argued that access to reproductive health is a human right and that assisted reproductive technologies (ARTs) should be accessible to all who need it, including couples with infertility living in low- and middle- income countries [6–8].

Alongside these developments, research on the lives of people with infertility in Sub-Saharan Africa (SSA) has flourished. Anthropological evidence and sociological research show how in several SSA countries, including The Gambia, parenthood and reproduction are important life projects, and having children is often considered a core dimension of one’s identity [9–11]. Consequences of infertility range from marital instability, marginalisation and stigmatisation by in-laws and community members to physical violence [7,9,12,13]. Women are particularly vulnerable to such repercussions because they are often perceived to be the ones responsible for fertility problems, regardless of any formal diagnosis of the underlying causes, which can be male-factor, female-factor, male-female factor or unknown [7,9,12].

Meanwhile, little is known about the perspectives and practices of different key stakeholders, here defined as individuals or organisations involved in political activities or professional care surrounding infertility [5]. When, for example, health care providers are interviewed, this tends to serve only for basic information about the medical care that is available [14]. A quick literature review indicates that the perspectives of other key stakeholders (e.g. international agencies, national governments, non-governmental organisations (NGOs) and individual advocates and activists) are largely absent when it comes to research on infertility [11]. This absence is problematic given the importance of involving a range of stakeholders in policy processes [15]. In practice, key decisions determining health system performance, including agenda setting, policy formulation and policy implementation, are often informed by the perspectives and behaviours of many of these stakeholders [4,15–17].

Understanding the perspectives of key stakeholders on infertility is particularly important in the context of SSA given the previously mentioned implications of infertility [18,19]. This is especially true in The Gambia, a small country in West Africa, which has, to date, hardly been the subject of research on health policy and systems [4,17]. The Gambia has recently faced major political change. The former authoritarian President Yahya Jammeh (in power between 1994–2017) was internationally criticised for his provision of herbal treatments to ‘cure’ infertility, HIV/AIDS and asthma [20–22]. His leadership was characterised by brutal treatment of his opponents, including targeted assassinations and forced exiles [21]. However, since early 2017, when a democratically elected government came into place following the 2016 elections and a brief transition period, there has been a re-opening of the broader health system to these issues, including an increased focus on infertility [20]. Furthermore, previous research on infertility in The Gambia has demonstrated the collaboration between organisations with a development agenda and *kanyaleng kafoolu* (i.e. organisations for women who have been unable to achieve the local reproductive norm of a large family) [3,20,23]. *Kanyaleng kafoo* membership serves both as a coping mechanism and as a proactive effort to overcome infertility or child mortality by begging God and confusing the spiritual forces (i.e. *kuntofengo* (a jinn) and *buwaa* (witchcraft)) perceived to cause infertility. Since the 1990s these groups have also been recruited by organisations to promote health and development messages because of their skills in song and dance [3,23]. Given the collaboration between *kanyalengs* and a diverse range of development organisations in The Gambia, an assessment of the experiences of stakeholders when it comes to infertility is potentially valuable.

This qualitative ethnographic study aims to explore the perspectives and practices from a broad range of key stakeholders regarding infertility in The Gambia and aims to address the following research questions: how do these key stakeholders in The Gambia understand and represent the needs and experiences of people living with infertility? What actions do these stakeholders take to improve the well-being of people living with infertility in The Gambia?

## Methods

### Research design

In-depth qualitative ethnographic research was carried out by SD and ME for a period of four months between September 2017 and August 2018 in the Greater Banjul area and the West Coast region of The Gambia. Primary data collection entailed the use of in-depth interviews, group discussions and participant observation, while sources of secondary data included government and NGO reports and media outputs.

### Study site, population and sampling

The West Coast region and Greater Banjul area were purposively chosen study sites as they are the most urbanised regions of the country hosting the central arm of the Ministry of Health and Social Welfare, the main referral hospital (Edward Francis Small Teaching Hospital) and most private health centres. The main campuses of the University of The Gambia and a multitude of policy makers and other key stakeholders such as those working in NGOs are located in these regions.

Women’s position in the country defies easy summary. Relative to men, women continue to have less access to economic resources and are less likely to complete their education [4]. However, The Gambia also has a number of women in parliament and many thriving organisations committed to improving the rights of women, mainly in the field of harmful cultural practices [4,24]. Several organisations are tackling the issue of female genital circumcision/mutilation (FGC/M), with type I (i.e. excision of the prepuce) and II (i.e. excision, clitoridectomy with partial or total exclusion of the labia minora) most prevalent in the country [24]. Yet, despite such efforts, and the laws against practices such as early marriages and FGC/M, these practices continue to take place [21].

There is a paucity of recent studies on infertility rates in The Gambia, although a contraceptive prevalence study conducted in 1993 found that around 2% of women at the end of their reproductive period had no children and considerably more women (11.6–15.2%) had a longer than expected time interval since their last birth [25]. A more recent study, published in 2001, reported 9.8% of participants to be infertile, with secondary infertility (defined as no pregnancy after at least 12 months of regular unprotected sexual intercourse) affecting 8.8% of all participants [26].

People were included in this study if they were willing to give informed written consent and came into contact with individuals with infertility given their professional background. In this study sample key stakeholders were formal health care providers, international agencies working on women’s rights and health, governmental institutions and NGOs working on the broader topic of women’s rights, infertility and/or who have been working with *kanyaleng kafoolu* (i.e. local groups of women who experience infertility and child loss and who organise themselves in order to cope with their circumstances and also to overcome their health problems) [3,20,27]. Some politicians and high-level policy-makers were contacted but were unfortunately unable to participate due to other obligations. The inclusion of different stakeholders was justified because infertility is not only a medical condition, but also very much a social and gendered problem impeding mainly women’s social and psychological well-being [9].

Participant recruitment was carried out through a mixed-sampling approach, both theoretical (based on research objectives and emergent findings) and snowball sampling techniques (relying on one person to contact the next person in the sample). Participant recruitment started before fieldwork, whereby the researchers contacted potential participants based on an internet search. During fieldwork participant recruitment was carried out continuously, through the theoretical sampling strategy and made use of snowball sampling, whereby respondents were either contacted (i) based on the network of the researchers or (ii) based on the referral of previous research participants.

### Positionality of the researchers

SD and ME carried out the empirical fieldwork; their positionality as white female researchers made them outsiders and might have had an effect on the answers. However, they are not complete outsiders as prior to carrying out this research, SD had resided in The Gambia (in total 14 months between 2012 and 2015). She was informally adopted by a local family and acquainted with culturally appropriate expressions and behaviour. ME position as a researcher with medical background and clinical experience was an asset in conducting research among health professionals.

### Data collection tools

#### Interviews

In total, 26 semi-structured interviews were conducted and audio-recorded upon consent, with stakeholders including health care providers (n = 11), stakeholders working for NGOs focussing on women’s rights in general or infertility specifically (n = 6), people working within governmental agencies (n = 8) and representatives of international organisations (n = 1) (Table 1). Interviews were conducted in English at places where respondents felt at ease, including their offices, meetings rooms, residencies or in public spaces such as restaurants and cafeterias (though still in relative privacy). Some informants were visited several times as an additional way of building trust and confidence. Interviews lasted approximately 60 minutes. Two question guides were developed, adjusted to people’s professional background (health care providers versus people working for relevant organisations) (S1 and S2 Files). These question guides were further adapted and refined according to emerging results. The question guides were used to structure the interviews but allowed for flexibility.

**Table 1:** Profile of respondents participating in interviews and informal conversations.

	Interviews (men/women)	Informal conversations (men/women)
Health care provider	11 (7/4)	6 (1/5)
Representative NGO	6 (0/6)	1 (0/1)
Representative governmental organisation	8 (6/2)	4 (2/2)
Representative international organisation	1 (1/0)	2 (1/1)
**TOTAL**	**26 (14/12)**	**13 (4/9)**

#### Participant observations and informal conversations

In total seven observations and 13 informal conversations were quickly jotted down on the spot or immediately afterwards, and elaborately written down at the earliest convenient time later that day (Table 1). Participant observation consisted of visiting several public and private health centres (at the following days: 5/10/2017; 27/11/2017; 28/11/2017; 31/01/2018; 06/02/2018; 06/04/2018) and observing activities organised by stakeholders which took place in public (e.g. a march for people with infertility on 14/10/2017). Informal conversations took place with health care providers (at the following days: 6/10/2017; 28/11/2017; 6/12/2017; 21/01/2018; 20/06/2018; 22/06/2018) and representatives of organisations (at the following days 2/10/2017; 22/11/2017). The researchers were always open about their identity as researchers during observations and informal conversations. These methods allow for a more contextualised understanding of the research questions. By establishing rapport and building trust these methods minimise socially desirable or otherwise biased answers.

#### Group discussions

In total, three group discussions were conducted because some informants expressed a desire to be interviewed together. The group discussions drew on the same question guide as was used in the interviews with stakeholders working for an organisation (S2 File). The first group discussion was conducted with three representatives of an NGO including a man and two women. The second group discussion involved three female representatives of a governmental organisation and the last group discussion took place with three female and three male representatives of an international organisation. Group discussions were carried out in private meeting rooms of organisations.

#### Secondary data

The study reviewed documents on infertility and gender published by the Gambian Government and local NGOs, and media outputs, including social media, before and during the period in the field [28,29,38–47,30,48–57,31,58–67,32,68–77,33,78–87,34–37]. The secondary data were used to inform and cross-check outcomes of the primary data analyses. These documents helped to generate a deeper understanding of social norms, the Gambian health system in general, and the availability of previous and current infertility treatments. They also provided a socio-political framework within which to further contextualise the findings.

### Data analysis

During data collection, the focus was both on process analysis (including reflexivity about positionality and the research process) and thematic content analysis. Thematic analysis was carried out by SD and ME and began with an immersion in the raw data by listening to audio recordings, reading and re-reading transcripts and secondary data. An open inductive coding approach was initially used to assign tentative codes, with themes emerging and being refined during this process. This process of mapping and interpreting results was influenced by the research objectives and the emergent themes in the data. The analysis of this data informed the question guides which were adapted based on initial results. The qualitative data analysis software programme NVivo (QSR International Pty Ltd. Version 11) was used to facilitate data organisation, retrieval of primary data and data analysis.

### Ethics

Ethical approval for this study was received from The Gambia Government/MRC Joint Ethics Committee (SCC1562), the ethical commissions of the Vrije Universiteit Brussel (ECHW_096), Belgium, and the Research Ethics Committee of The University of Sheffield School of Health and Related Research (ScHARR) (018955), United Kingdom. The interviewers followed the Code of Ethics of the American Anthropological Association (AAA). People were informed about project goals, the topic and type of questions as well as their right to decline participation or to interrupt the conversation at any time. Anonymity was guaranteed and confidentiality assured. Written informed consent was obtained before each interview, which was documented with a signature. Participants were offered a copy of the information and consent document. Anonymity was guaranteed, and confidentiality assured by using only descriptive identifiers and assigning a unique code to each data source. In addition, to ensure anonymity, the gender and profession of the respondents is not mentioned in the quotes on the more sensitive topics related to the political context. For the same reason no distinction was made in the quotes from representatives of NGOs working on women’s rights in general and actors working on infertility specifically.

## Results

### Stakeholders’ understandings and representations of infertility in The Gambia

#### Awareness about the financial, social and emotional impact of infertility

The accounts of all stakeholders on the implications of infertility reflected the stories shared by women living with infertility (see [9] for details on the experiences of women with infertility). There was a general recognition that–regardless of any diagnosis–broader society often perceived infertility to be a woman’s problem and the consequences of infertility were much more severe for women than men. Some respondents also believed that infertility is much more common among women.

‘*This scenario happened to a family member*: *he married this girl for five years without having a child*. *Then the family members said*: *“this woman will never have a child*, *and this man needs a child*. *So*, *the best thing we could do is it to have another wife”*. *The other wife came and she also spends five years without a child*. *Those two wives are there without a child*. *The second one said*: *“I cannot sit and take no action”*. *Just recently*, *he divorced*. *I said to my family*: *“it is not the cause of the women*, *it is our relative”*. *They said*: *“no you are lying*, *he is a man”*. *So*, *the blame is on the woman not the man*.’

(Women representing NGO, group discussion)

Several health care providers reflected on the unrealistic expectations set upon married couples regarding childbearing, whereby societal norms prescribe that women should become pregnant within the first few months following marriage. Yet, respondents largely did not question this strong pro-natal norm in The Gambia, as it was said to be self-evident that marriage should lead to children. The topic of infertility remains a taboo in Gambian society and several stakeholders acknowledged the stigmatisation of people living with infertility, with much of the focus on women. They explained how infertility often leads to marital and financial problems. Several male stakeholders put forward the role of the extended family pressuring their son or brother to engage in polygyny when a wife does not become pregnant.

‘*If a woman is married here in Africa without getting a child*, *they blame the lady and she may feel very inconvenient*, *uncomfortable in the compound*. *The sisters*, *the extended family members will gossip about her*, *and they will even prompt the husband to go and look for another wife*. *Even me*, *that was my problem*, *after three years of being married without getting a child my mind said*: *“let me look for another wife”*.’

(Male health care provider, interview)

A respondent who had previously conducted research on sex work explained that during his research he came across several female sex workers who were infertile. Following divorce, these women were pushed into prostitution, because their families had refused to take them back into the compound, leaving them with no place to live nor resources to rely on.

#### Awareness of treatment seeking among people living with infertility

Stakeholders’ understandings of the causes and treatments of infertility did not differ greatly from the stories of women with infertility (see [20] for details on people’s treatment seeking in case of infertility). They mentioned that many individuals rely on indigenous healers and attend sacred places for treatment. Several stakeholders, including health care providers, stated that they did not find this problematic, although health care providers reported wanting patients to attend biomedical health care facilities first. Illustrative of the compatibility of clinical and indigenous frameworks is the position and story of a male nurse who also worked as an indigenous healer:

‘*My wife*, *I am telling you*, *she had two operations*. *The first operation*, *the medical doctor said the fallopian tubes were blocked and they flushed it*. *She lost one of the fallopian tubes*, *so she was left with one*. *After two years also*, *they say she has a cyst*. *She was operated to no avail*. *Until 2011*, *when I was doing my bachelor in nursing then I started practicing black medicine [i*.*e*. *spiritual treatment for infertility]*. *She was one of the first patients that I started with*. *At the beginning of 2012*, *she got her first pregnancy*.’

(Male health care provider, interview)

Stakeholders stressed that women often seek treatment alone, with little or no support from their husbands. Health care providers reported having to appeal to the husbands to join their wives and to be diagnosed themselves, often to no avail. Most stakeholders discussed how women living in rural areas of the country are more vulnerable compared to urban women, since there are severe limitations in information and services related to sexual and reproductive health inland. Private health centres are perceived to offer the best available treatment, however for most people they are unaffordable and very difficult to reach. Health care providers were often critical about the quality of care provided by other health care providers working in both private and/or public health sectors in the urban area. They were mainly concerned that the provided treatment was ineffective and/or insufficiently monitored. Health care providers working in either private or public sector also criticised the global inequalities in the availability of infertility services. They described often having to refer patients in need of in vitro fertilisation (IVF) to clinics abroad (specifically Ghana, India, Spain or England), while being aware that most patients could not afford this treatment, nor the associated travel costs.

#### Positions and actions of stakeholders in relation to infertility in The Gambia

While no distinction could be made regarding respondents’ awareness about infertility, stakeholders diverged in their positions on what to do about the issue, if anything. Based on their reported and apparent patterns of engagement, we identified three clusters of stakeholders, namely; (i) stakeholders specifically focusing on raising awareness of, and addressing, infertility, who can be described as ‘infertility supporters’; (ii) stakeholders indirectly working on infertility by addressing some of the perceived causes of infertility, who we have labelled ‘infertility moderate supporters’; and (iii) stakeholders who are aware of the issues but do not consider infertility to be a current priority, namely the ‘infertility neutrals or moderate opponents’. Overall, the largest portion of study participants were infertility moderate supporters, followed by the infertility neutrals and moderate opponents and then the infertility supporters. After providing some insights into these three distinct positions, we discuss how the national context influences the capacities of infertility supporters and the tensions and opportunities that exist between these contrasting positions.

#### Three different positions in the public domain

*Position 1*: *infertility supporters*. This study identified three stakeholders working specifically on infertility, championing the cause. One is an NGO set-up by a Gambian woman outside her regular working hours, indicating her strong motivation to place infertility high on the public agenda. The organisation has a two-fold objective: (i) to improve access to biomedical treatment for people who find it hard to conceive or who have a history of repeated miscarriages; and (ii) to fight against the stigma associated with infertility. Although the organisation is relatively young, observations and interviews indicated that many activities have already been carried out with the aim of raising awareness, reducing stigma and placing infertility on the public agenda in The Gambia. These activities include: (i) a march attended by approximately 350 persons (predominantly women) located in the centre of the urban area; (ii) the creation and broadcasting of radio jingles in Mandinka, Wolof and English to provide information on infertility treatment and to protest against the stigmatisation of women living with infertility; and (iii) frequent active use of social media channels to spread information on reproductive health, biomedical causes and treatments and other activities around the globe related to infertility. Although difficult to quantify, these actions appear to have impact, with social media posts frequently ‘liked’ and ‘shared’. Numerous individuals have also contacted the organisation to discuss their personal problems. Despite the absence of funding, and a lack of formal health or social care training, the founder aims to support fellow Gambians affected by infertility wherever possible.

The last two stakeholders working on infertility are two health care providers. Based on their professional experiences, these health care providers identified a need for affordable and technologically advanced treatment services. The first health care provider contacted several organisations in Europe in order to bring better treatment options to The Gambia, but to little avail. However, he was still eager to collaborate and find new partnerships so that better treatment could be provided. The second health care provider has a specialisation in infertility treatment and is a major player in the provision of treatment services who founded an organisation to address the issue of infertility in The Gambia. Primary and secondary data show that this organisation has currently two objectives, namely: (i) to develop sensitisation programs on infertility prevention; and (ii) to build partnerships and generate funding with companies and organisations working on infertility abroad in order to improve local infertility treatment services. In the practice, the aim is to provide both affordable treatment and counselling though it was mentioned that she can fall short when it comes to counselling because she does not have professional training or adequate support in this area.

These three stakeholders highlighted how both women and men can have infertility problems and need to be involved in treatment seeking. They were enthusiastic about the current activism concerning infertility, however, at the time the research was conducted cooperation seemed to be limited.

*Position 2*: *infertility moderate supporters*. There are a multitude of organisations who are not directly working on infertility, but who associate infertility with some of their organisational goals, particularly providing comprehensive sexual education and reducing harmful cultural practices.

These stakeholders expect that sexual education could prevent a range of conditions that may precipitate or contribute to the prevalence of infertility, including sexually transmitted infections (STIs) and subsequent pelvic inflammatory disease (PID). The limited use of condoms leading to STIs is not only caused by the lack of comprehensive sexual education, but also limited availability of condoms, especially in rural areas. Furthermore, it is considered immoral to engage in sexual relationships, let alone be pregnant, outside marriage. To avoid shame and stigmatisation, women will illegally engage in unsafe and unhygienic abortions which also cause infertility:

*‘Some of the women become pregnant because they are not taking family planning commodities and they might end up not wanting that pregnancy or trying to get rid of it*. *In this country it is not legally accepted to do an abortion*. *You have a lot of implications*: *even the site*, *the instruments*, *the procedure*, *could all be catastrophic*. *In that regard*, *it might also have some side effects; some may experience infertility problems as a result of that procedure many years after it was done because you don’t want to have kids out of wedlock*.’

(Man representing international organisation, group discussion)

Several governmental organisations and NGOs associated infertility with harmful cultural practices. The first harmful cultural practice many stakeholders associated with infertility was FGC/M. They argued that (i) infibulation complicates deliveries leading to higher chances of secondary infertility (i.e. infertility after having been able to become pregnant previously) and (ii) type I and II of FGC/M could also lead to infections of the fallopian tubes and the womb, ultimately making women infertile. The stakeholders perceived the cutting itself to be problematic, as well as the healing process including the application of herbs and sometimes animal faeces, leading to high risks of infections. They reported that these problems were mentioned during community sensitisations in order to convince people to stop the practice:

‘*If the circumciser cut you deeply*, *you will have infertility problems*. *You will have very serious problems*. *So*, *we link these two together during sensitisations to make them understand*: *yes*, *it is important*, *it is our culture*, *we appreciate culture very well*, *but let’s eradicate culture that is affecting us*.’

(Women representing NGO, group discussion)

In contrast, some health care providers questioned whether FGC/M had any influence on the fertility of women, mentioning that most Gambian women are circumcised and only few are unable to become pregnant.

Another harmful cultural practice that was perceived to be a risk factor for infertility is child marriage. Pro-natal norms put married girls and women under pressure to have children regardless of the maturity of the female body. According to several participants, fistula—which can cause infertility—is common among young women who have an early first pregnancy, especially in rural areas where women often deliver at home. Many stakeholders specifically asked for more research on the association between these harmful cultural practices and infertility, stressing that in order to convince people of these links, it is important to have information based on research conducted within the Gambian context.

*Position 3*: *infertility neutrals and moderate opponents*. The final group of stakeholders did not consider infertility a priority. A stakeholder representing an international organisation working on sexual and reproductive health stated that given the high fertility rate of 5.8 children in the country, their organisation disregarded infertility and continued to focus on family planning. Although he described the choice to reproduce as a right, he thought it unlikely that his organisation would work on this topic in the future in The Gambia or across SSA. Most health care providers did not consider infertility a priority given the resource constraints within the health system. Several health care providers identified a need to improve basic services (e.g. anaesthetics) before more specialised infertility services could be provided. Media reports suggest several problems existing in the national health system as health care providers held a month-long strike in March 2018 in response to criticism by the former Minister of Health. Health care providers situated infertility within a context where a plethora of infectious and non-communicable diseases affect the population with a greater perceived degree of urgency and importance.

#### Extremely limited funding for infertility and high competition in a resource-limited setting

A salient feature constraining the activism of stakeholders, according to both primary and secondary data sources, is donor dependency. Several stakeholders acknowledged that due to this dependency they could not always follow their own priorities. In practice, a balance is often struck between, on the one hand, the demands from donors and, on the other hand, organisational objectives. Infertility supporters were sometimes funded with the help of local sponsors and partners (e.g. a local bank), but were also often self-funded through out of pocket expenditures. It was explained that the ability to materialise future plans (including amongst others organising support groups, making a documentary and setting up a fund so people who are financially vulnerable can go for medical treatment), largely depends on donations and the opportunities that might arise from building stronger national and international networks.

#### A new political climate and opportunities for reproductive activism and engagement

Respondents advocating for people living with infertility situated their activism within the new political climate. During the time former President Yahya Jammeh was in power, infertility was considered to be a politically sensitive topic, since the president had his own herbal treatment and there was looming threat of eviction and imprisonment for those who interfered.

*Yahya Jammeh had his own program*. *I don’t know if he did it on Tuesday or Thursday morning but he had his own fertility program […] So*, *for me*, *to come out and say ‘I am here’*, *I was a little bit careful in that*. *It is only now that I am being more open about the health services for people with infertility I am providing*.

(Interview)

*I have been in contact with organisation X providing infertility services*, *but they were cautious in the past because the previous president said he could treat infertility so they did not want to come*.

(Interview)

*The first person who said anything about the herbal treatment of the previous president was send out of this country*. *So*, *you can imagine ordinary Gambians when they want to say something*, *what will come out of it*? *Director X of an international organisation Y was asked to leave this country because he said what the guy [i*.*e*. *previous president Yahya Jammeh] was doing with HIV was wrong*.

(Interview)

The quotes above show that most stakeholders were critical of the herbal treatment provided by the previous president. According to some stakeholders, including infertility supporters, infertility is not considered a priority for the current government as there are ‘*much bigger problems’*. However, infertility supporters hope that in the future the lives of women with infertility would improve. This optimism resulted partly from news that the current First Lady Fatoumatta Bah Barrow is working together with an international organisation raising awareness about infertility. Secondary data showed how she has, to date, supported the training on infertility care and treatment of two Gambian doctors in India, who have since returned to practice in The Gambia.

## Discussion

This study provides a rich overview of stakeholders’ understandings and representations of infertility, and their heterogeneous practices to improve the well-being of people living with infertility in The Gambia.

Stakeholders’ understandings of the impact of infertility and treatment-seeking behaviour is firmly located in the shared social and cultural world of people living with infertility in The Gambia. This is important as understanding the cultural frameworks of patients and their social realities are identified as a prerequisite for the provision of culturally competent and congruent care [15,88]. Though most stakeholders claimed to accept indigenous healing methods, they also argued that people should first attend formal biomedical health services. Stakeholders were aware that this was challenging, particularly for impoverished people and those living in rural areas. This study has also shown that stakeholders had a good understanding of the impact of infertility on the lives of women, but that they knew less about the experiences of men with infertility. This can be explained by the gendered, socio-cultural context of infertility: women’s identity and status in a society marked by gender inequality and pro-natal norms are very closely tied to childbearing and motherhood [9,89]. Policymakers could facilitate behaviour change and create a supportive social context for people with infertility by implementing awareness campaigns to reduce the stigma surrounding the condition and to improve societal knowledge relating to male-factor infertility. These campaigns should not only target the women themselves but also their husbands, their families and the broader community.

Health care providers formulated concerns about the quality of treatment services their colleagues offered. A review by Gerrits & Shaw [2] shows that throughout SSA infertility services within the public health sector are incomplete, rudimentary and unsystematically provided. Additionally, several health care providers were critical about the global inequalities in access to reproductive technologies. Despite high prevalence rates of infertility in the SSA region, international political commitments and the many technical advances when it comes to infertility treatment, there are still a limited number of health centres where reproductive technology is available in SSA. In The Gambia, health care providers allocated the limited availability and quality of a broad range of treatment services for infertility to the many life-threatening diseases in a resource constrained health system. Consequentially, many of them did not consider the provision of infertility services to be a priority. This rationale is not unique for Gambian health care providers as similar arguments have been made in other SSA countries and by the international community [2,8,11,90]. Yet, inequalities in access to infertility services–a right enshrined 25 years ago during the Cairo conference and Program of Action–keep Gambian women trapped in a cycle of shame and poor health.

In The Gambia, several stakeholders have initiated activities to support people living with infertility despite contextual difficulties. This illustrates the agency of community members when it comes to health. They cannot be portrayed as passive recipients following donor interests. This analysis therefore favours a sociological approach of agency instead of a more limited and decontextualized psychological conceptualisation of agency as the exercise of individual choice [16,91]. This sociological approach allows us to understand how the social and political context enables or limits choices in specific settings. Hence, the agency of these stakeholders should be contextualised within the recent change in political leadership and governance in The Gambia which has enabled stakeholders to set-up organisations working on infertility. These organisations aim to reduce the stigma surrounding infertility, increase knowledge about reproductive health and improve access to better treatment and counselling services. At the time of the research, infertility supporters aim to bring much needed treatment services to people with infertility in collaboration with private health centres in The Gambia [20]. This is something the Gambian government–and many other governments around the world–currently still largely neglects, despite the ICPD’s Program of Action recommendation that treatment and prevention of infertility should be integrated into broader reproductive health services. However, there is a tendency throughout SSA, and indeed worldwide, to locate infertility treatment services within the private health sector, leading to the question of how accessible these treatment services are for more disadvantaged populations [92,93].

The difficulties stakeholders working on infertility face in attracting funds should not be interpreted as merely an administrative problem. The limited availability, affordability and quality of infertility treatment services in The Gambia–as in many other SSA countries–can be explained by the limited interest of international donors but also shortages in national human and economic resources, the existence of other health priorities and concerns about overpopulation [8,20,94]. These arguments are also put forward by many health care providers and a representative of an international organisation working in The Gambia who did not perceive infertility to be a priority. The position of the health care providers towards infertility results from existing barrier in providing care; they are struggling to work under severe resource constraints and pressures within the borders of the current health care system [4,17]. However, the position of these health care providers and the broader global health community triggers important ethical discussions about who receives (or fails to receive) care and what kind of health issues are deemed important and which ones are not deemed important.

We would like to discuss the position of many stakeholders working on comprehensive sexual education and harmful cultural practices in the country. Opinions about FGC/M as a plausible cause of infertility differed among stakeholders. Within the broader literature, there is considerable debate surrounding the role of FGC/M in infertility. Previous research conducted in the North Bank Region, surrounding Farafenni–a transport hub located in a rural area of The Gambia–did not find any linkages between type II of FGC/M and infertility [95]. As Morison et al. [95] indicate, this does not mean that there is no association at all, since infertility can occur if pelvic infection causes damage to reproductive organs. It merely implies that given the limited sample size, they found no association between infertility and type II FGC/M in that particular setting. When it comes to child marriages and infertility, studies remain unclear about the possible relationship. Multiple studies have shown how child marriages (i.e. defined as married below the age of 18 years) can have detrimental health and social consequences for girls and young women [96]. Child marriages are associated with coerced sexual intercourse and symptoms of genital tract infections which, if left untreated, may lead to infertility. Furthermore, young mothers experience higher risk of obstructed labour, postpartum haemorrhage and sepsis [96–98] which might lead to secondary infertility. Interestingly, stakeholders did not mention how women with infertility are more likely to be confronted with polygynous marriages [9,12]. This can be explained by the highly religious normative framing of polygyny in The Gambia making it a difficult topic to contest in public. Following the call from various stakeholders, additional research is essential to better understand possible associations between harmful cultural practices and infertility in the country.

Previous research has shown that major underlying factors of infertility problems in SSA are preventable conditions such as untreated STIs, postpartum infections and unsafe abortions [2]. Walraven et al. [26] found that reproductive health problems are considered to be normal and commonplace in The Gambia. It was reported how women often did not discuss these problems unless directly questioned by health workers, therefore it is expected that comprehensive sexual and reproductive health education could create more awareness and even prevent a range of conditions that may precipitate or contribute to the prevalence of infertility, such as STIs, unsafe and unhygienic deliveries and abortions [26,95]. The government should invest in the early detection and appropriate treatment of STIs, and pregnancy- and abortion-related sepsis [14,92]. While it might not be feasible to provide technologically-advanced infertility treatments services in the short term, it has been shown in other SSA countries that major improvements in basic infertility care can be achieved at low cost and relative ease by standardising diagnosis and treatment procedures, training health staff and improving counselling practices at all levels of the health care system [2,99].

## Conclusion

This work showcases the positions and perspectives on infertility of a range of stakeholders, including health care providers, NGOs, governmental agencies and international organisations working in The Gambia. The voices of these stakeholders have previously been largely omitted from research on infertility, yet they are important mediators in the responsiveness of health systems to the needs of people living with infertility. Stakeholders generally showed an understanding of the daily challenges of infertility. Despite the many obstacles faced, several stakeholders have endeavoured to champion the importance of infertility, engaging to improve the well-being of people living with infertility. This demonstrates their agency, which we argue needs to be documented out of respect for their struggles and because stakeholders’ perspectives and practices should be considered when developing services to improve the lives of people with infertility. These infertility supporters aim to bring infertility services to the community, a task the national government and international donors are still largely neglecting. Infertility supporters must remain aware of the views and perspectives of many health care providers who do not consider infertility a priority. Working to overcome such constraints will require infertility supporters to sensitise and collaborate with this group of stakeholders, as well as with other organisations working in The Gambia, as they are crucial actors in the provision of services for people with infertility. At the policy level, investments should be made in awareness campaigns to reduce stigma and promote attendance to the health centres for reproductive health challenges. Additionally, major improvements in infertility care can be achieved at low cost by implementing standardised diagnosis and treatment procedures, through further training of health staff and via the development of counselling practices at all levels of the health care system.

## Supporting information

S1 File Question guide health care providers. (PDF)

S2 File Question guide stakeholders working for an organisation. (PDF)
