# Supplementary material for: The fertile grounds of reproductive activism in The Gambia: A qualitative study of local key stakeholders’ understandings and heterogeneous actions related to infertility
Source: PLoS One. 2019 Dec 4;14(12):e0226079. doi: 10.1371/journal.pone.0226079 (PMC6892487; doi:10.1371/journal.pone.0226079)
Supplement: S1 File — (PDF) [file pone.0226079.s001.pdf]

Question guide interviews and group discussions with health care providers

Background health care provider

Could you tell me more about the kind of work you do? What is your role?

How long have you been in this role?

Which services do you provide?

Are you involved with any sexual or reproductive health programs or services?

Do you see sexual or reproductive health patients?

Probe: can you give me any examples?

Do you ever see any male patients seeking sexual and reproductive health services?

Probe: can you give me any examples?

Experiences with people with infertility

Do you sometimes see people with a fertility problem at your consultations?

Probe: how often do you see patients with a fertility problem?

Probe: what kind of problems do they have?

Probe: do you see cases of primary infertility, where couples struggle to have any children at all?

Probe: do you see any cases of secondary infertility, where couples have a child or children, but struggle to have additional children?

Probe: are those mostly men, women, or do couples come together?

Probe: what is the most common profile of people attending you in terms of background, education, age?

If no, probe: are you aware of these kinds of situations?

Could you tell me about these consultations, what happens at them?

What do the patients usually tell you about the reasons they come and see you?

What do you tell them at the consultation?

Are there any non-medical problems you are faced with when trying to help patients with fertility problems?

Probe: can you give me an example?

Have you had any special training for dealing with infertility patients?

(If yes, probe:) what kind of?

Probe: where?

Information about health center

How often is the health center where you provide services out of service?

Is there enough electricity/water?

Is there enough health personal? What is their profile?

Is there enough medication available?

Which area does this health center cover? How many villages?

Is there a computerized or systematic record system within and between hospitals?

If not, probe: could this imply that clients visit multiple sources of health care and go through the same procedures several times?

Explanations of infertility

Why do you think some couples cannot have children here in The Gambia?

Prompt with: STIs, FGM, PCOS (are clomiphene/metformin available?), multiple partners)

How does the initial problem lead to problems conceiving?

What do your patients / the general public think is the cause of their infertility?

Many people perceive fibroids to be the cause of their infertility problems. Could

you explain your experiences and perspective on this?

Another reason people often mention is seketoo. Do patients ever come to you with this problem?

Could you tell me a little bit more about buluntoo?

Do you ever experience that your patients have a different opinion about the cause of their infertility to you?

If yes, probe: how did you notice?

Do you know of any traditional or cultural beliefs about the causes of infertility?

How do you personally feel about these? How do you combine them with your medical understanding of the causes of infertility?

Care provision

What sexual health and infertility health services are offered here?

Can you describe the services?

What treatments are offered?

How do people access the services? Do they have to be referred?

Do you know which medical solutions are offered for people with fertility problems in The Gambia?

Probe: can you describe the services?

Probe: where can people find this kind of treatment?

Are services available for men as well as women?

Probe: what sort of services/treatments do women receive?

Probe: what sort of services/treatments do men receive?

How do you in general try to help infertile patients?

Do patients usually accept your advice?

(If not, probe:) can you give me an example of a case in which a patient did not accept your advice?

Do you have any idea why patients sometimes do not accept your advice?

How do your patients usually feel about what you offer them?

Probe: are they satisfied with what you offer them?

Probe: can you tell me about a time that that was the case?

Probe: are they also disappointed sometimes?

Probe: can you tell me about a time that that was the case?

Infertility – barriers and opportunities in access to care in The Gambia

What do you feel are the main barriers to people attending infertility clinics/services /accessing treatment?

How might we be able to overcome this barrier?

Prompt with: issues around training, availability staff

Do you think that people's interpretation of seketoo could be a barrier to going to the biomedical health sector? In which way?

What do you think are the main facilitators/opportunities that encourage people to access infertility services?

Prompt with: The First Lady's interest in infertility, NGOs

Relationship with other healers

Do your patients also seek other solutions than consulting you?

Probe: what kind of?

Probe: at what phase in their life do they visit these other healers?

Probe: do you think people prefer traditional approaches for infertility?

Why do you think people do this?

What do you think about that?

Do you know what indigenous healers do for people with an infertility problem?

Do you refer your patients to traditional healers?

Socio-emotional impacts of infertility upon patients in The Gambia

What do you feel is the impact of infertility?

Does it affect men or women more?

Are there psychological problems too? Are you aware of it causing depression?

Is there a cultural or social impact? Such as individuals becoming isolated or outcast?

Are there any financial impacts of infertility?

Are there any legal impacts (such as implications for inheritance and marriage)?

Are you aware of any support groups for those affected by infertility?

Women and men facing reproductive challenges are often stigmatised, do you or your hospital do something to minimalize the stigma?

Training

Did you receive any teaching on sexual health, fertility and infertility whilst training at university or school?

How much teaching did you receive and at which stages of training?

What was covered in these sessions?

Probe: anatomy, physiology, pathology, psychological/social impact

Did you cover male infertility?

If yes, probe: what did you learn about?

Did you find this teaching useful?

At the time, did you think it was an important topic to learn about?

What were your main learning points or take away messages from these sessions?

Have you had any further teaching or training on the topic of infertility at work? (Such as CPD sessions) and has this furthered your knowledge?

Do you feel confident and comfortable having conversations about fertility and infertility based on your training?

Based on your experience now, did you feel anything was lacking in the training?

Do you feel that your patients may need more psychological support?

Do you feel you would like to have additional teaching or training on sexual and reproductive health? If so, specifically what?

Awareness

Does the public have a good understanding of fertility and infertility?

Do you think fertility is an important issue in society?

Do you think infertility is a priority?

Government support

Are you aware of any national or international guidelines for infertility?

Given that The Gambia is a country with limited resources and high levels of fertility overall, do you think that infertility should be a priority for the government?

Probe: why?

Does the government support you in any way? How?

Probe: what are according to you the current priorities of the government?

Closing

Is there anything further, that I have not asked you, that you feel is important or would like to tell me?

Do you have any questions for me?
