# Supplementary material for: The fertile grounds of reproductive activism in The Gambia: A qualitative study of local key stakeholders’ understandings and heterogeneous actions related to infertility
Source: PLoS One. 2019 Dec 4;14(12):e0226079. doi: 10.1371/journal.pone.0226079 (PMC6892487; doi:10.1371/journal.pone.0226079)
Supplement: S2 File — (PDF) [file pone.0226079.s002.pdf]

Question guide interviews and group discussions with stakeholders working for an organisation

Personal background

What is your function within this organization?

What are your responsibilities?

How long do you already work for this organization?

What is your background?

Background organization

What are the objectives of your organization?

How does your organization try to realise these objectives?

> *If they organize sensitization, probe:* which messages are convincing (e.g. health, rights?)
>
> *If they talk about education:* what does this entail?

Does your organization also engage itself to improve the lives of men, how?

What is your organization trying to achieve currently?

Does your organization work on the topic of infertility?

> *Probe:* why?
>
> *Probe:* what do you do?

Has your organization worked on the topic of infertility in the past?

Do you feel a lot has changed for women the last ten years in The Gambia?

> *Probe:* which changes do you notice?

Knowledge about infertility

Why do people in The Gambia want to have children?

What are the causes of infertility?

Probe: what is the role of witches, jinni?

Probe: what is the role of abortions?

Where do people with infertility go for treatment?

Probe: is there a difference between ethnic groups?

Probe: do you know of any other places people go to?

Probe: why do you think people go to indigenous healers?

Probe: where are they likely to go first, and afterwards?

Which treatment options are available at these places?

What do you think about the quality of these treatment services?

What do you think about the biomedical health care provisions in general?

What are the involved costs for these different treatment options?

What is an important barrier for people to go for biomedical treatment?

What are the consequences of infertility (women versus men)? Are certain people more vulnerable, who?

Probe: what about divorce?

Probe: what is the impact of the relationship with the family-in-law?

Probe: what is the impact on the relationship of people with the broader community?

Probe: what are the financial consequences?

Do you think it is possible to have a happy life without children in The Gambia?

How do people cope with the challenges of infertility?

Is there a lot of attention in public space to the topic of infertility?

Collaboration between organization and kanyalengs

Is your organization in contact with women with infertility?

If the organization works together with kanyalengs, probe:

- how is it decide with which groups you work together with?
- how does the collaboration with kanyalengs take shape?

What is the influence of the cooperation between organizations and kanyalengs, on the practice of kanyalengs?

How does the cooperation influence the social status of kanyalengs?

Kanyalengs

When do women become kanyalengs?

Probe: are women sometimes forced to become members?

Why are certain women with infertility kanyalengs and others not?

Are kanyalengs considered to be modern?

What is the role of kanyalengs?

What do they do during gatherings?

Probe: what is the function of these gatherings?

What are the advantages of being a kanyaleng?

Why do kanyaleng women sometimes have strange names?

What is the attitude of men towards kanyalengs?

What do you think about kanyalengs?

If the organization is targeting harmful cultural practices

Many organizations describe child and forced marriages, female genital circumcision and polygyny as harmful cultural practices, what do you think about this?

Probe: do you consider these practices as cultural/religious?

Do you see any association between infertility and harmful cultural practices?

If yes, probe: which ones?

If yes, probe: do you take this idea along in your sensitization campaigns?

Do you see any associations between reproductive health and harmful cultural practices?

If yes, probe: which ones?

If yes, probe: do you take this idea along in your sensitization campaigns?

If the organization is targeting child marriages:

When are girls considered to be ready for marriage in Gambian culture?

Probe: are there any difference between ethnic groups?

What do you understand to be child marriages?

Probe: do you make any distinction between child and early marriage?

Probe: how do you see the gender difference in child marriages?

What is the current situation when it comes to child marriages in urban Gambia,

Why do you think people engage in child marriages?

Are child marriages always negative?

Probe: what in case of consent?

Role of government

Do you think the current government is doing enough to help people with infertility?

Do you think the current government is doing enough effort to enforce the laws on harmful cultural practices?

How do you see the changes with the new government in comparison with the government of Jammeh?

Probe: are there any differences in how people talk about rights?

Closing

Is there anything further, that I have not asked you, that you feel is important or would like to tell me?

Do you have any questions for me?
